# Trichloridobis(ethyl­diphenyl­phosphine)(tetra­hydro­furan)­molybdenum(III)

**DOI:** 10.1107/S1600536810021690

**Published:** 2010-06-16

**Authors:** Tomasz Kruczyński, Jerzy Pikies, Łukasz Ponikiewski

**Affiliations:** a Chemical Faculty, Gdańsk University of Technology, Narutowicza 11/12, Gdańsk PL 80233, Poland

## Abstract

In the mononuclear title compound, [MoCl_3_(C_4_H_8_O)(C_14_H_15_P)_2_], obtained by the reaction of trichloro­tris­(tetra­hydro­furan)­molybdenum(III) and ethyl­diphenyl­phosphine in tetra­hydro­furan (THF) solution, the Mo^III^ atom is six-coordinated by one O atom of a THF mol­ecule, two P atoms from two ethyl­diphenyl­phosphine ligands and three Cl atoms in a distorted octa­hedral geometry. The C atoms of the THF molecule are disordered over two positions in a 0.55 (2):0.45 (2) ratio.

## Related literature

For the structures of similar molybdenum complexes and for bond-length data, see: Cotton & Jianrui (1996 ▶); Cotton & Vidyasagar (1995 ▶); Hofacker *et al.* (1989 ▶); Borgmann *et al.* (1997 ▶). For the synthesis, see: Anker *et al.* (1975 ▶).
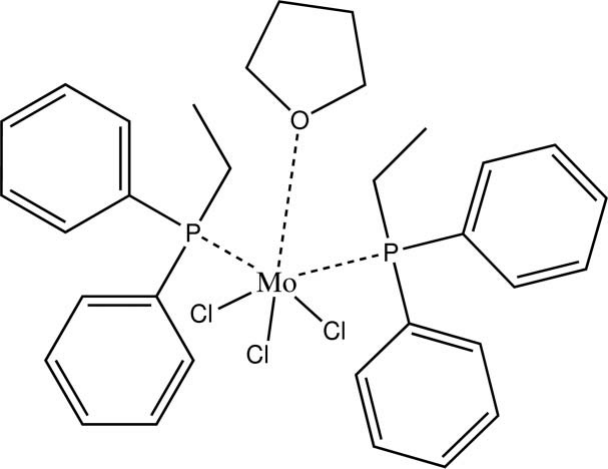


## Experimental

### 

#### Crystal data

[MoCl_3_(C_4_H_8_O)(C_14_H_15_P)_2_]
*M* *_r_* = 702.85
Monoclinic, 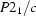

*a* = 15.437 Å
*b* = 13.356 Å
*c* = 20.229 Å
β = 128.87°
*V* = 3247.1 Å^3^
*Z* = 4
Mo *K*α radiation
μ = 0.77 mm^−1^
*T* = 120 K
0.13 × 0.10 × 0.04 mm

#### Data collection

Oxford Diffraction KM-4/Xcalibur diffractometer with a Sapphire2 detector
Absorption correction: analytical [*CrysAlis RED* (Oxford Diffraction, 2006 ▶). based on expressions derived by Clark & Reid (1995 ▶)] *T*_min_ = 0.937, *T*_max_ = 0.970
25240 measured reflections
7022 independent reflections
2985 reflections with *I* > 2σ(*I*)
*R*_int_ = 0.089

#### Refinement

*R*[*F* ^2^ > 2σ(*F* ^2^)] = 0.044
*wR*(*F* ^2^) = 0.115
*S* = 0.81
7022 reflections
385 parameters
H-atom parameters constrained
Δρ_max_ = 0.70 e Å^−3^
Δρ_min_ = −0.54 e Å^−3^

### 

Data collection: *CrysAlis CCD* (Oxford Diffraction, 2006 ▶); cell refinement: *CrysAlis RED* (Oxford Diffraction, 2006 ▶); data reduction: *CrysAlis RED*; program(s) used to solve structure: *SHELXS97* (Sheldrick, 2008 ▶); program(s) used to refine structure: *SHELXL97* (Sheldrick, 2008 ▶); molecular graphics: *ORTEP-3 for Windows* (Farrugia, 1997 ▶); software used to prepare material for publication: *WinGX* (Farrugia, 1999 ▶).

## Supplementary Material

Crystal structure: contains datablocks global, I. DOI: 10.1107/S1600536810021690/ez2211sup1.cif

Structure factors: contains datablocks I. DOI: 10.1107/S1600536810021690/ez2211Isup2.hkl

Additional supplementary materials:  crystallographic information; 3D view; checkCIF report

## Figures and Tables

**Table 1:** Selected bond lengths (Å)

Mo1—O1	2.206 (3)
Mo1—Cl1	2.3871 (13)
Mo1—Cl2	2.3822 (13)
Mo1—Cl3	2.4126 (13)
Mo1—P1	2.5964 (14)
Mo1—P2	2.5974 (13)

**Fig. 1.**
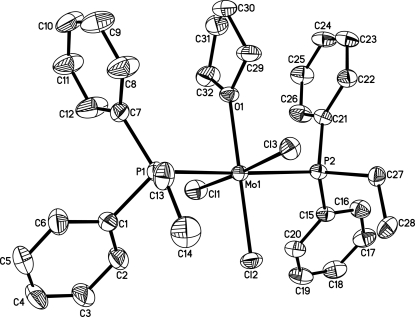
The molecular structure of the title compound showing the atomic numbering scheme. Displacement ellipsoids are drawn at the 50% probability level. For clarity, all H atoms and the minor component of the disorder have been omitted.

## supplementary crystallographic information

### Comment

The title molecule [MoCl_3_(PEtPh_2_)(THF)] was prepared as a potential adduct for synthesis with lithium phosphanides of the formula *R*_2_P—P(SiMe_3_)Li (*R* = *^t^*Bu, ^i^Pr, Et_2_N, ^i^Pr_2_N).

The Mo^III^ atom resides in a distorted MoCl_3_OP_2_ octahedral environment. The equatorial positions are occupied by three Cl atoms and one O atom from the THF, while the axial positions are occupied by P atoms from two ethyldiphenylphosphine residues. The Mo—Cl bond length [2.3871 (13) Å, 2.3822 (13) Å, 2.4126 (13) Å], the Mo—P bond length [2.5964 (14) Å, 2.5974 (13) Å] and the Mo—O bond length [2.206 (3) Å] are very similar to the previously reported molybdenum complexes (Cotton & Vidyasagar, 1995; Cotton & Jianrui, 1996; Hofacker *et al.*, 1989; Borgmann *et al.*,  1997).

Atoms C29, C30, C31, C32 from the THF molecule were disordered over two positions. During the refinement process the disorder models were refined with occupancies of 0.55 (2) and 0.45 (2).

### Experimental

The title compound was prepared according to the previously published method (Anker *et al.*, 1975)

### Refinement

Atoms C29, C30, C31, C32 were disordered over two positions. During the refinement process the disorder models were refined with occupancies of 0.55 (2) and 0.45 (2). H atoms bonded to C were included in calculated positions and refined as riding on their parent C atom with C—H = 0.95 Å, *U*_iso_(H) = 1.2 *U*_eq_(C) for aromatic; C—H = 0.99 Å, *U*_iso_(H) = 1.2 *U*_eq_(C) for methylene; and C—H = 0.98 Å, *U*_iso_(H) = 1.5 *U*_eq_(C) for methyl atoms.

### Crystal data

**Table:**

[MoCl_3_(C_4_H_8_O)(C_14_H_15_P)_2_]	*F*(000) = 1444
*M**_r_* = 702.85	*D*_x_ = 1.438 Mg m^−^^3^
Monoclinic, *P*2_1_/*c*	Mo *K*α radiation, λ = 0.71073 Å
Hall symbol: -P 2ybc	Cell parameters from 5220 reflections
*a* = 15.437 Å	θ = 2.0–28.9°
*b* = 13.356 Å	µ = 0.77 mm^−^^1^
*c* = 20.229 Å	*T* = 120 K
β = 128.87°	Block, orange
*V* = 3247.1 Å^3^	0.13 × 0.1 × 0.04 mm
*Z* = 4	

### Data collection

**Table:**

Oxford Diffraction KM-4/Xcalibur diffractometer with a Sapphire2 (large Be window) detector	7022 independent reflections
graphite	2985 reflections with *I* > 2σ(*I*)
Detector resolution: 8.1883 pixels mm^-1^	*R*_int_ = 0.089
ω scans	θ_max_ = 27°, θ_min_ = 2°
Absorption correction: analytical [*CrysAlis RED* (Oxford Diffraction, 2006). based on expressions derived by Clark & Reid (1995)]	*h* = −15→19
*T*_min_ = 0.937, *T*_max_ = 0.97	*k* = −16→17
25240 measured reflections	*l* = −25→22

### Refinement

**Table:**

Refinement on *F*^2^	Primary atom site location: structure-invariant direct methods
Least-squares matrix: full	Secondary atom site location: difference Fourier map
*R*[*F*^2^ > 2σ(*F*^2^)] = 0.044	Hydrogen site location: inferred from neighbouring sites
*wR*(*F*^2^) = 0.115	H-atom parameters constrained
*S* = 0.81	*w* = 1/[σ^2^(*F*_o_^2^) + (0.0551*P*)^2^] where *P* = (*F*_o_^2^ + 2*F*_c_^2^)/3
7022 reflections	(Δ/σ)_max_ = 0.001
385 parameters	Δρ_max_ = 0.70 e Å^−^^3^
0 restraints	Δρ_min_ = −0.54 e Å^−^^3^

### Special details

**Table:**

Experimental. CrysAlis RED (Oxford Diffraction, 2006) Analytical numeric absorption correction using a multifaceted crystal model based on expressions derived by Clark & Reid (1995)
Geometry. All e.s.d.'s (except the e.s.d. in the dihedral angle between two l.s. planes) are estimated using the full covariance matrix. The cell e.s.d.'s are taken into account individually in the estimation of e.s.d.'s in distances, angles and torsion angles; correlations between e.s.d.'s in cell parameters are only used when they are defined by crystal symmetry. An approximate (isotropic) treatment of cell e.s.d.'s is used for estimating e.s.d.'s involving l.s. planes.
Refinement. Refinement of *F*^2^ against ALL reflections. The weighted *R*-factor *wR* and goodness of fit *S* are based on *F*^2^, conventional *R*-factors *R* are based on *F*, with *F* set to zero for negative *F*^2^. The threshold expression of *F*^2^ > σ(*F*^2^) is used only for calculating *R*-factors(gt) *etc*. and is not relevant to the choice of reflections for refinement. *R*-factors based on *F*^2^ are statistically about twice as large as those based on *F*, and *R*- factors based on ALL data will be even larger.

### Fractional atomic coordinates and isotropic or equivalent isotropic displacement parameters (Å^2^)

**Table:**

	*x*	*y*	*z*	*U*_iso_*/*U*_eq_	Occ. (<1)
Mo1	0.57979 (3)	0.74957 (4)	0.63166 (2)	0.03356 (12)	
Cl1	0.53421 (13)	0.92350 (10)	0.61183 (10)	0.0612 (4)	
Cl2	0.62891 (9)	0.74919 (11)	0.76928 (7)	0.0511 (3)	
Cl3	0.62090 (10)	0.57325 (9)	0.64269 (9)	0.0488 (3)	
P1	0.78469 (10)	0.78721 (10)	0.69759 (9)	0.0415 (3)	
P2	0.37606 (10)	0.70228 (10)	0.56343 (8)	0.0370 (3)	
O1	0.5361 (2)	0.7484 (3)	0.50482 (18)	0.0411 (7)	
C1	0.8589 (4)	0.8883 (4)	0.7753 (3)	0.0495 (14)	
C2	0.8104 (5)	0.9421 (5)	0.8018 (4)	0.075 (2)	
H2A	0.7358	0.9277	0.7785	0.09*	
C3	0.8682 (6)	1.0174 (6)	0.8621 (5)	0.099 (3)	
H3A	0.8334	1.0547	0.8797	0.118*	
C4	0.9750 (6)	1.0370 (5)	0.8957 (4)	0.084 (2)	
H4A	1.0161	1.0871	0.938	0.101*	
C5	1.0232 (5)	0.9849 (5)	0.8687 (4)	0.0711 (19)	
H5A	1.0976	1	0.8918	0.085*	
C6	0.9674 (4)	0.9118 (4)	0.8095 (4)	0.0645 (17)	
H6A	1.0026	0.8765	0.7913	0.077*	
C7	0.8016 (4)	0.8198 (4)	0.6187 (3)	0.0462 (13)	
C8	0.8262 (6)	0.7500 (6)	0.5828 (4)	0.0873 (15)	
H8A	0.8389	0.6826	0.6018	0.105*	
C9	0.8329 (5)	0.7744 (6)	0.5206 (4)	0.084 (2)	
H9A	0.8507	0.7242	0.4977	0.101*	
C10	0.8145 (5)	0.8694 (6)	0.4914 (4)	0.080 (2)	
H10A	0.8208	0.8866	0.449	0.096*	
C11	0.7871 (6)	0.9387 (5)	0.5234 (4)	0.0873 (15)	
H11A	0.7712	1.0052	0.5021	0.105*	
C12	0.7820 (7)	0.9141 (5)	0.5872 (5)	0.098 (2)	
H12A	0.7641	0.9649	0.6097	0.117*	
C13	0.8800 (4)	0.6808 (4)	0.7548 (4)	0.0563 (16)	
H13A	0.85	0.6221	0.7166	0.068*	
H13B	0.9529	0.6977	0.7698	0.068*	
C14	0.8976 (5)	0.6530 (5)	0.8342 (4)	0.084 (2)	
H14A	0.9562	0.6022	0.8654	0.126*	
H14B	0.8283	0.6261	0.8194	0.126*	
H14C	0.9198	0.7126	0.87	0.126*	
C15	0.2980 (4)	0.7756 (3)	0.5873 (3)	0.0414 (13)	
C16	0.1881 (4)	0.7565 (4)	0.5442 (3)	0.0572 (14)	
H16A	0.1526	0.705	0.5026	0.069*	
C17	0.1273 (5)	0.8118 (5)	0.5608 (4)	0.0656 (17)	
H17A	0.051	0.7972	0.5318	0.079*	
C18	0.1780 (5)	0.8869 (5)	0.6187 (4)	0.0639 (17)	
H18A	0.1364	0.9264	0.6291	0.077*	
C19	0.2870 (5)	0.9059 (5)	0.6615 (4)	0.0675 (18)	
H19A	0.3217	0.9584	0.7021	0.081*	
C20	0.3483 (4)	0.8502 (4)	0.6471 (3)	0.0524 (14)	
H20A	0.4255	0.8633	0.6785	0.063*	
C21	0.2853 (4)	0.7107 (4)	0.4475 (3)	0.0416 (13)	
C22	0.2670 (4)	0.6302 (4)	0.3982 (3)	0.0497 (14)	
H22A	0.2983	0.567	0.4241	0.06*	
C23	0.2041 (4)	0.6398 (5)	0.3121 (3)	0.0594 (16)	
H23A	0.1927	0.5835	0.2787	0.071*	
C24	0.1579 (4)	0.7294 (5)	0.2740 (3)	0.0630 (18)	
H24A	0.1137	0.7357	0.2141	0.076*	
C25	0.1750 (4)	0.8081 (5)	0.3209 (4)	0.0621 (16)	
H25A	0.1427	0.8707	0.2942	0.075*	
C26	0.2390 (4)	0.8001 (4)	0.4079 (4)	0.0599 (16)	
H26A	0.2509	0.8574	0.4405	0.072*	
C27	0.3571 (4)	0.5747 (4)	0.5836 (3)	0.0466 (13)	
H27A	0.2765	0.5598	0.5468	0.056*	
H27B	0.3913	0.5281	0.5678	0.056*	
C28	0.4074 (4)	0.5557 (4)	0.6752 (3)	0.0624 (16)	
H28A	0.3979	0.485	0.6824	0.094*	
H28B	0.3699	0.5977	0.6901	0.094*	
H28C	0.4868	0.5721	0.7123	0.094*	
C29	0.542 (4)	0.6602 (15)	0.4672 (19)	0.064 (3)	0.55 (2)
H29A	0.4819	0.6128	0.4502	0.077*	0.55 (2)
H29B	0.6146	0.6263	0.5077	0.077*	0.55 (2)
C30	0.5284 (16)	0.6963 (11)	0.3911 (10)	0.066 (3)	0.55 (2)
H30A	0.4919	0.6449	0.3458	0.079*	0.55 (2)
H30B	0.6012	0.7139	0.4062	0.079*	0.55 (2)
C31	0.4556 (16)	0.7877 (16)	0.3635 (13)	0.073 (5)	0.55 (2)
H31A	0.4697	0.8363	0.3343	0.088*	0.55 (2)
H31B	0.3758	0.7691	0.325	0.088*	0.55 (2)
C32	0.488 (5)	0.8294 (17)	0.4431 (19)	0.068 (7)	0.55 (2)
H32A	0.5427	0.8838	0.4634	0.081*	0.55 (2)
H32B	0.4218	0.857	0.4344	0.081*	0.55 (2)
C29A	0.541 (5)	0.6650 (18)	0.461 (2)	0.056 (3)	0.45 (2)
H29C	0.5087	0.604	0.4656	0.067*	0.45 (2)
H29D	0.6191	0.6509	0.4857	0.067*	0.45 (2)
C30A	0.474 (2)	0.6981 (14)	0.3695 (13)	0.075 (4)	0.45 (2)
H30C	0.4962	0.6617	0.3397	0.091*	0.45 (2)
H30D	0.393	0.6895	0.3382	0.091*	0.45 (2)
C31A	0.506 (2)	0.8069 (16)	0.3820 (17)	0.063 (4)	0.45 (2)
H31C	0.4502	0.8462	0.3304	0.076*	0.45 (2)
H31D	0.5797	0.8153	0.3959	0.076*	0.45 (2)
C32A	0.510 (6)	0.839 (2)	0.455 (3)	0.064 (7)	0.45 (2)
H32C	0.5683	0.8906	0.4901	0.077*	0.45 (2)
H32D	0.4375	0.867	0.4346	0.077*	0.45 (2)

### Atomic displacement parameters (Å^2^)

**Table:**

	*U*^11^	*U*^22^	*U*^33^	*U*^12^	*U*^13^	*U*^23^
Mo1	0.0421 (2)	0.0281 (2)	0.0318 (2)	0.00186 (18)	0.02382 (17)	0.0012 (2)
Cl1	0.0978 (11)	0.0283 (8)	0.0685 (10)	0.0094 (7)	0.0575 (9)	0.0041 (7)
Cl2	0.0565 (7)	0.0651 (8)	0.0343 (6)	−0.0004 (7)	0.0297 (6)	0.0013 (8)
Cl3	0.0583 (8)	0.0320 (7)	0.0577 (9)	0.0070 (6)	0.0372 (7)	0.0059 (6)
P1	0.0426 (8)	0.0390 (8)	0.0446 (8)	−0.0019 (5)	0.0281 (7)	−0.0007 (6)
P2	0.0405 (7)	0.0363 (8)	0.0323 (8)	0.0025 (5)	0.0220 (6)	−0.0010 (6)
O1	0.0568 (19)	0.0352 (18)	0.0328 (16)	0.0028 (17)	0.0288 (15)	0.0022 (19)
C1	0.057 (4)	0.048 (4)	0.045 (3)	−0.008 (2)	0.032 (3)	−0.005 (3)
C2	0.076 (4)	0.081 (5)	0.086 (5)	−0.033 (3)	0.060 (4)	−0.042 (4)
C3	0.109 (6)	0.114 (6)	0.105 (6)	−0.055 (5)	0.083 (5)	−0.069 (5)
C4	0.088 (5)	0.074 (5)	0.069 (5)	−0.040 (4)	0.039 (4)	−0.031 (4)
C5	0.055 (4)	0.062 (5)	0.075 (5)	−0.018 (3)	0.031 (4)	−0.012 (4)
C6	0.060 (4)	0.057 (4)	0.080 (5)	−0.013 (3)	0.046 (4)	−0.011 (3)
C7	0.049 (3)	0.046 (4)	0.053 (3)	−0.004 (2)	0.036 (3)	−0.004 (3)
C8	0.154 (5)	0.060 (3)	0.093 (4)	0.013 (3)	0.099 (4)	0.013 (3)
C9	0.100 (5)	0.096 (6)	0.086 (5)	0.038 (4)	0.073 (4)	0.009 (4)
C10	0.114 (5)	0.085 (6)	0.089 (5)	0.006 (4)	0.087 (5)	0.007 (4)
C11	0.154 (5)	0.060 (3)	0.093 (4)	0.013 (3)	0.099 (4)	0.013 (3)
C12	0.204 (8)	0.041 (4)	0.116 (6)	0.005 (4)	0.133 (6)	0.006 (4)
C13	0.048 (3)	0.049 (4)	0.066 (4)	0.003 (2)	0.033 (3)	0.014 (3)
C14	0.074 (4)	0.084 (5)	0.067 (5)	0.013 (3)	0.031 (4)	0.029 (4)
C15	0.046 (3)	0.042 (4)	0.033 (3)	0.007 (2)	0.024 (3)	0.002 (2)
C16	0.051 (3)	0.067 (4)	0.057 (3)	0.003 (3)	0.036 (3)	−0.008 (3)
C17	0.058 (4)	0.086 (5)	0.064 (4)	0.018 (3)	0.044 (4)	0.009 (4)
C18	0.072 (4)	0.069 (5)	0.061 (4)	0.028 (3)	0.047 (4)	0.010 (3)
C19	0.079 (5)	0.070 (5)	0.056 (4)	0.011 (3)	0.044 (4)	−0.011 (3)
C20	0.051 (3)	0.061 (4)	0.042 (3)	0.004 (3)	0.028 (3)	−0.005 (3)
C21	0.040 (3)	0.044 (3)	0.036 (3)	0.002 (2)	0.022 (3)	−0.006 (2)
C22	0.054 (3)	0.050 (4)	0.048 (3)	0.005 (2)	0.033 (3)	0.001 (3)
C23	0.060 (4)	0.072 (5)	0.035 (3)	0.003 (3)	0.024 (3)	−0.012 (3)
C24	0.056 (4)	0.089 (5)	0.030 (3)	0.013 (3)	0.020 (3)	−0.005 (3)
C25	0.067 (4)	0.061 (4)	0.047 (4)	0.022 (3)	0.030 (3)	0.013 (3)
C26	0.076 (4)	0.047 (4)	0.047 (4)	0.014 (3)	0.034 (3)	0.002 (3)
C27	0.051 (3)	0.040 (3)	0.052 (3)	−0.004 (2)	0.034 (3)	0.001 (3)
C28	0.077 (4)	0.059 (4)	0.058 (4)	−0.004 (3)	0.045 (3)	0.014 (3)
C29	0.089 (5)	0.066 (4)	0.053 (5)	−0.004 (4)	0.052 (5)	−0.013 (4)
C30	0.084 (6)	0.076 (5)	0.053 (6)	−0.004 (5)	0.051 (6)	−0.011 (4)
C31	0.086 (14)	0.083 (10)	0.040 (9)	−0.014 (9)	0.035 (11)	0.004 (7)
C32	0.094 (19)	0.057 (6)	0.049 (8)	0.010 (8)	0.043 (12)	0.020 (6)
C29A	0.086 (5)	0.055 (5)	0.052 (5)	−0.005 (4)	0.056 (5)	−0.012 (4)
C30A	0.094 (7)	0.077 (6)	0.058 (6)	−0.003 (6)	0.049 (6)	−0.014 (5)
C31A	0.076 (14)	0.085 (10)	0.036 (10)	−0.011 (9)	0.039 (12)	0.003 (7)
C32A	0.092 (19)	0.055 (6)	0.047 (8)	0.008 (8)	0.044 (12)	0.020 (6)

### Geometric parameters (Å, °)

**Table:**

Mo1—O1	2.206 (3)	C16—H16A	0.95
Mo1—Cl1	2.3871 (13)	C17—C18	1.356 (8)
Mo1—Cl2	2.3822 (13)	C17—H17A	0.95
Mo1—Cl3	2.4126 (13)	C18—C19	1.348 (7)
Mo1—P1	2.5964 (14)	C18—H18A	0.95
Mo1—P2	2.5974 (13)	C19—C20	1.371 (7)
P1—C7	1.824 (6)	C19—H19A	0.95
P1—C1	1.830 (5)	C20—H20A	0.95
P1—C13	1.840 (5)	C21—C26	1.364 (7)
P2—C27	1.818 (5)	C21—C22	1.370 (7)
P2—C21	1.830 (5)	C22—C23	1.368 (7)
P2—C15	1.838 (5)	C22—H22A	0.95
O1—C29	1.434 (15)	C23—C24	1.358 (8)
O1—C29A	1.453 (16)	C23—H23A	0.95
O1—C32	1.455 (15)	C24—C25	1.326 (7)
O1—C32A	1.459 (17)	C24—H24A	0.95
C1—C2	1.366 (7)	C25—C26	1.380 (7)
C1—C6	1.389 (7)	C25—H25A	0.95
C2—C3	1.390 (8)	C26—H26A	0.95
C2—H2A	0.95	C27—C28	1.514 (7)
C3—C4	1.358 (9)	C27—H27A	0.99
C3—H3A	0.95	C27—H27B	0.99
C4—C5	1.359 (9)	C28—H28A	0.98
C4—H4A	0.95	C28—H28B	0.98
C5—C6	1.355 (8)	C28—H28C	0.98
C5—H5A	0.95	C29—C30	1.499 (17)
C6—H6A	0.95	C29—H29A	0.99
C7—C12	1.358 (8)	C29—H29B	0.99
C7—C8	1.373 (8)	C30—C31	1.510 (16)
C8—C9	1.364 (9)	C30—H30A	0.99
C8—H8A	0.95	C30—H30B	0.99
C9—C10	1.352 (9)	C31—C32	1.464 (16)
C9—H9A	0.95	C31—H31A	0.99
C10—C11	1.341 (8)	C31—H31B	0.99
C10—H10A	0.95	C32—H32A	0.99
C11—C12	1.381 (9)	C32—H32B	0.99
C11—H11A	0.95	C29A—C30A	1.514 (18)
C12—H12A	0.95	C29A—H29C	0.99
C13—C14	1.498 (8)	C29A—H29D	0.99
C13—H13A	0.99	C30A—C31A	1.506 (17)
C13—H13B	0.99	C30A—H30C	0.99
C14—H14A	0.98	C30A—H30D	0.99
C14—H14B	0.98	C31A—C32A	1.509 (18)
C14—H14C	0.98	C31A—H31C	0.99
C15—C16	1.359 (7)	C31A—H31D	0.99
C15—C20	1.371 (7)	C32A—H32C	0.99
C16—C17	1.391 (7)	C32A—H32D	0.99
			
O1—Mo1—Cl2	179.21 (10)	C18—C17—H17A	120.3
O1—Mo1—Cl1	88.22 (10)	C16—C17—H17A	120.3
Cl2—Mo1—Cl1	92.44 (5)	C19—C18—C17	120.3 (5)
O1—Mo1—Cl3	88.34 (10)	C19—C18—H18A	119.8
Cl2—Mo1—Cl3	91.01 (5)	C17—C18—H18A	119.8
Cl1—Mo1—Cl3	176.50 (6)	C18—C19—C20	120.8 (6)
O1—Mo1—P1	89.05 (9)	C18—C19—H19A	119.6
Cl2—Mo1—P1	90.49 (5)	C20—C19—H19A	119.6
Cl1—Mo1—P1	91.92 (5)	C19—C20—C15	119.8 (5)
Cl3—Mo1—P1	88.66 (4)	C19—C20—H20A	120.1
O1—Mo1—P2	89.76 (8)	C15—C20—H20A	120.1
Cl2—Mo1—P2	90.67 (4)	C26—C21—C22	117.8 (5)
Cl1—Mo1—P2	90.94 (5)	C26—C21—P2	120.3 (4)
Cl3—Mo1—P2	88.41 (4)	C22—C21—P2	121.8 (4)
P1—Mo1—P2	176.87 (5)	C23—C22—C21	120.8 (5)
C7—P1—C1	102.7 (2)	C23—C22—H22A	119.6
C7—P1—C13	103.8 (3)	C21—C22—H22A	119.6
C1—P1—C13	101.9 (3)	C24—C23—C22	120.4 (6)
C7—P1—Mo1	113.20 (16)	C24—C23—H23A	119.8
C1—P1—Mo1	119.23 (19)	C22—C23—H23A	119.8
C13—P1—Mo1	114.15 (18)	C25—C24—C23	119.6 (6)
C27—P2—C21	104.1 (2)	C25—C24—H24A	120.2
C27—P2—C15	102.5 (2)	C23—C24—H24A	120.2
C21—P2—C15	101.7 (2)	C24—C25—C26	120.9 (6)
C27—P2—Mo1	114.84 (16)	C24—C25—H25A	119.6
C21—P2—Mo1	111.36 (16)	C26—C25—H25A	119.6
C15—P2—Mo1	120.40 (17)	C21—C26—C25	120.6 (6)
C29—O1—C32	108.9 (15)	C21—C26—H26A	119.7
C29A—O1—C32	104.0 (14)	C25—C26—H26A	119.7
C29—O1—C32A	113.6 (15)	C28—C27—P2	113.6 (4)
C29A—O1—C32A	108.5 (16)	C28—C27—H27A	108.8
C29—O1—Mo1	123.2 (9)	P2—C27—H27A	108.8
C29A—O1—Mo1	128.2 (10)	C28—C27—H27B	108.8
C32—O1—Mo1	127.6 (11)	P2—C27—H27B	108.8
C32A—O1—Mo1	122.9 (12)	H27A—C27—H27B	107.7
C2—C1—C6	118.3 (5)	C27—C28—H28A	109.5
C2—C1—P1	121.5 (4)	C27—C28—H28B	109.5
C6—C1—P1	120.2 (4)	H28A—C28—H28B	109.5
C1—C2—C3	121.1 (6)	C27—C28—H28C	109.5
C1—C2—H2A	119.5	H28A—C28—H28C	109.5
C3—C2—H2A	119.5	H28B—C28—H28C	109.5
C4—C3—C2	119.2 (7)	O1—C29—C30	105.5 (14)
C4—C3—H3A	120.4	O1—C29—H29A	110.6
C2—C3—H3A	120.4	C30—C29—H29A	110.6
C5—C4—C3	120.0 (6)	O1—C29—H29B	110.6
C5—C4—H4A	120	C30—C29—H29B	110.6
C3—C4—H4A	120	H29A—C29—H29B	108.8
C6—C5—C4	121.3 (6)	C29—C30—C31	103.3 (18)
C6—C5—H5A	119.3	C29—C30—H30A	111.1
C4—C5—H5A	119.3	C31—C30—H30A	111.1
C5—C6—C1	120.1 (6)	C29—C30—H30B	111.1
C5—C6—H6A	120	C31—C30—H30B	111.1
C1—C6—H6A	120	H30A—C30—H30B	109.1
C12—C7—C8	115.8 (6)	C32—C31—C30	104.0 (19)
C12—C7—P1	121.3 (5)	C32—C31—H31A	111
C8—C7—P1	122.7 (4)	C30—C31—H31A	111
C9—C8—C7	122.1 (7)	C32—C31—H31B	111
C9—C8—H8A	118.9	C30—C31—H31B	111
C7—C8—H8A	118.9	H31A—C31—H31B	109
C10—C9—C8	120.7 (7)	O1—C32—C31	107.4 (17)
C10—C9—H9A	119.7	O1—C32—H32A	110.2
C8—C9—H9A	119.7	C31—C32—H32A	110.2
C11—C10—C9	118.7 (7)	O1—C32—H32B	110.2
C11—C10—H10A	120.7	C31—C32—H32B	110.2
C9—C10—H10A	120.7	H32A—C32—H32B	108.5
C10—C11—C12	120.4 (6)	O1—C29A—C30A	105.3 (18)
C10—C11—H11A	119.8	O1—C29A—H29C	110.7
C12—C11—H11A	119.8	C30A—C29A—H29C	110.7
C7—C12—C11	122.2 (7)	O1—C29A—H29D	110.7
C7—C12—H12A	118.9	C30A—C29A—H29D	110.7
C11—C12—H12A	118.9	H29C—C29A—H29D	108.8
C14—C13—P1	113.7 (4)	C31A—C30A—C29A	100 (2)
C14—C13—H13A	108.8	C31A—C30A—H30C	111.8
P1—C13—H13A	108.8	C29A—C30A—H30C	111.8
C14—C13—H13B	108.8	C31A—C30A—H30D	111.8
P1—C13—H13B	108.8	C29A—C30A—H30D	111.8
H13A—C13—H13B	107.7	H30C—C30A—H30D	109.5
C13—C14—H14A	109.5	C30A—C31A—C32A	104 (2)
C13—C14—H14B	109.5	C30A—C31A—H31C	110.9
H14A—C14—H14B	109.5	C32A—C31A—H31C	110.9
C13—C14—H14C	109.5	C30A—C31A—H31D	110.9
H14A—C14—H14C	109.5	C32A—C31A—H31D	110.9
H14B—C14—H14C	109.5	H31C—C31A—H31D	108.9
C16—C15—C20	119.4 (5)	O1—C32A—C31A	105 (2)
C16—C15—P2	119.5 (4)	O1—C32A—H32C	110.7
C20—C15—P2	121.1 (4)	C31A—C32A—H32C	110.7
C15—C16—C17	120.3 (6)	O1—C32A—H32D	110.7
C15—C16—H16A	119.8	C31A—C32A—H32D	110.7
C17—C16—H16A	119.8	H32C—C32A—H32D	108.8
C18—C17—C16	119.4 (6)		
			
O1—Mo1—P1—C7	11.0 (2)	P1—C7—C8—C9	176.5 (5)
Cl2—Mo1—P1—C7	−169.7 (2)	C7—C8—C9—C10	−0.5 (12)
Cl1—Mo1—P1—C7	−77.2 (2)	C8—C9—C10—C11	−1.4 (11)
Cl3—Mo1—P1—C7	99.3 (2)	C9—C10—C11—C12	2.3 (11)
O1—Mo1—P1—C1	131.9 (2)	C8—C7—C12—C11	−0.5 (11)
Cl2—Mo1—P1—C1	−48.8 (2)	P1—C7—C12—C11	−175.6 (6)
Cl1—Mo1—P1—C1	43.7 (2)	C10—C11—C12—C7	−1.4 (12)
Cl3—Mo1—P1—C1	−139.8 (2)	C7—P1—C13—C14	168.8 (4)
O1—Mo1—P1—C13	−107.4 (2)	C1—P1—C13—C14	62.4 (5)
Cl2—Mo1—P1—C13	71.9 (2)	Mo1—P1—C13—C14	−67.6 (5)
Cl1—Mo1—P1—C13	164.4 (2)	C27—P2—C15—C16	−58.1 (5)
Cl3—Mo1—P1—C13	−19.1 (2)	C21—P2—C15—C16	49.4 (5)
O1—Mo1—P2—C27	108.1 (2)	Mo1—P2—C15—C16	173.0 (4)
Cl2—Mo1—P2—C27	−71.3 (2)	C27—P2—C15—C20	122.9 (4)
Cl1—Mo1—P2—C27	−163.7 (2)	C21—P2—C15—C20	−129.7 (4)
Cl3—Mo1—P2—C27	19.7 (2)	Mo1—P2—C15—C20	−6.1 (5)
O1—Mo1—P2—C21	−9.9 (2)	C20—C15—C16—C17	−0.2 (8)
Cl2—Mo1—P2—C21	170.76 (18)	P2—C15—C16—C17	−179.3 (4)
Cl1—Mo1—P2—C21	78.31 (18)	C15—C16—C17—C18	1.8 (9)
Cl3—Mo1—P2—C21	−98.26 (18)	C16—C17—C18—C19	−1.9 (9)
O1—Mo1—P2—C15	−128.8 (2)	C17—C18—C19—C20	0.3 (9)
Cl2—Mo1—P2—C15	51.90 (18)	C18—C19—C20—C15	1.3 (9)
Cl1—Mo1—P2—C15	−40.55 (18)	C16—C15—C20—C19	−1.3 (8)
Cl3—Mo1—P2—C15	142.89 (18)	P2—C15—C20—C19	177.8 (4)
Cl1—Mo1—O1—C29	−180 (2)	C27—P2—C21—C26	150.8 (5)
Cl3—Mo1—O1—C29	0(2)	C15—P2—C21—C26	44.6 (5)
P1—Mo1—O1—C29	89 (2)	Mo1—P2—C21—C26	−84.9 (5)
P2—Mo1—O1—C29	−89 (2)	C27—P2—C21—C22	−33.3 (5)
Cl1—Mo1—O1—C29A	179 (3)	C15—P2—C21—C22	−139.5 (4)
Cl3—Mo1—O1—C29A	−2(3)	Mo1—P2—C21—C22	91.0 (4)
P1—Mo1—O1—C29A	87 (3)	C26—C21—C22—C23	−0.1 (8)
P2—Mo1—O1—C29A	−90 (3)	P2—C21—C22—C23	−176.1 (4)
Cl1—Mo1—O1—C32	−6(3)	C21—C22—C23—C24	−0.6 (9)
Cl3—Mo1—O1—C32	173 (3)	C22—C23—C24—C25	0.7 (9)
P1—Mo1—O1—C32	−98 (3)	C23—C24—C25—C26	−0.1 (9)
P2—Mo1—O1—C32	85 (3)	C22—C21—C26—C25	0.7 (8)
Cl1—Mo1—O1—C32A	7(4)	P2—C21—C26—C25	176.7 (4)
Cl3—Mo1—O1—C32A	−173 (4)	C24—C25—C26—C21	−0.6 (9)
P1—Mo1—O1—C32A	−85 (4)	C21—P2—C27—C28	−169.0 (4)
P2—Mo1—O1—C32A	98 (4)	C15—P2—C27—C28	−63.3 (4)
C7—P1—C1—C2	128.5 (5)	Mo1—P2—C27—C28	69.0 (4)
C13—P1—C1—C2	−124.3 (5)	C32—O1—C29—C30	17 (4)
Mo1—P1—C1—C2	2.4 (6)	C32A—O1—C29—C30	5(5)
C7—P1—C1—C6	−52.0 (5)	Mo1—O1—C29—C30	−168.8 (15)
C13—P1—C1—C6	55.3 (5)	O1—C29—C30—C31	−30 (4)
Mo1—P1—C1—C6	−178.0 (4)	C29—C30—C31—C32	33 (4)
C6—C1—C2—C3	−0.9 (10)	C29—O1—C32—C31	4(5)
P1—C1—C2—C3	178.7 (6)	C29A—O1—C32—C31	6(5)
C1—C2—C3—C4	−0.6 (12)	C32A—O1—C32—C31	120 (18)
C2—C3—C4—C5	1.6 (12)	Mo1—O1—C32—C31	−170.0 (17)
C3—C4—C5—C6	−1.2 (11)	C30—C31—C32—O1	−23 (4)
C4—C5—C6—C1	−0.3 (10)	C32—O1—C29A—C30A	−11 (4)
C2—C1—C6—C5	1.3 (9)	C32A—O1—C29A—C30A	−22 (5)
P1—C1—C6—C5	−178.2 (5)	Mo1—O1—C29A—C30A	165.2 (15)
C1—P1—C7—C12	−47.7 (6)	O1—C29A—C30A—C31A	38 (4)
C13—P1—C7—C12	−153.6 (5)	C29A—C30A—C31A—C32A	−39 (4)
Mo1—P1—C7—C12	82.1 (5)	C29—O1—C32A—C31A	−4(6)
C1—P1—C7—C8	137.5 (5)	C29A—O1—C32A—C31A	−3(6)
C13—P1—C7—C8	31.6 (6)	C32—O1—C32A—C31A	−73 (13)
Mo1—P1—C7—C8	−92.7 (5)	Mo1—O1—C32A—C31A	170 (2)
C12—C7—C8—C9	1.4 (10)	C30A—C31A—C32A—O1	27 (5)
